# Meat, the metabolites: an integrated metabolite profiling and lipidomics approach for the detection of the adulteration of beef with pork

**DOI:** 10.1039/c6an00108d

**Published:** 2016-02-25

**Authors:** Drupad K. Trivedi, Katherine A. Hollywood, Nicholas J. W. Rattray, Holli Ward, Dakshat K. Trivedi, Joseph Greenwood, David I. Ellis, Royston Goodacre

**Affiliations:** a Manchester Institute of Biotechnology (MIB) , School of Chemistry , University of Manchester , 131 Princess Street , Manchester , M1 7DN , UK . Email: roy.goodacre@manchester.ac.uk; b Faculty of Life Sciences , University of Manchester , Manchester , M13 9PL , UK

## Abstract

Adulteration of high quality food products with sub-standard and cheaper grades is a world-wide problem taxing the global economy. Currently, many traditional tests suffer from poor specificity, highly complex outputs and a lack of high-throughput processing. Metabolomics has been successfully used as an accurate discriminatory technique in a number of applications including microbiology, cancer research and environmental studies and certain types of food fraud. In this study, we have developed metabolomics as a technique to assess the adulteration of meat as an improvement on current methods. Different grades of beef mince and pork mince, purchased from a national retail outlet were combined in a number of percentage ratios and analysed using GC-MS and UHPLC-MS. These techniques were chosen because GC-MS enables investigations of metabolites involved in primary metabolism whilst UHPLC-MS using reversed phase chromatography provides information on lipophilic species. With the application of chemometrics and statistical analyses, a panel of differential metabolites were found for identification of each of the two meat types. Additionally, correlation was observed between metabolite content and percentage of fat declared on meat products’ labelling.

## Abstract 2

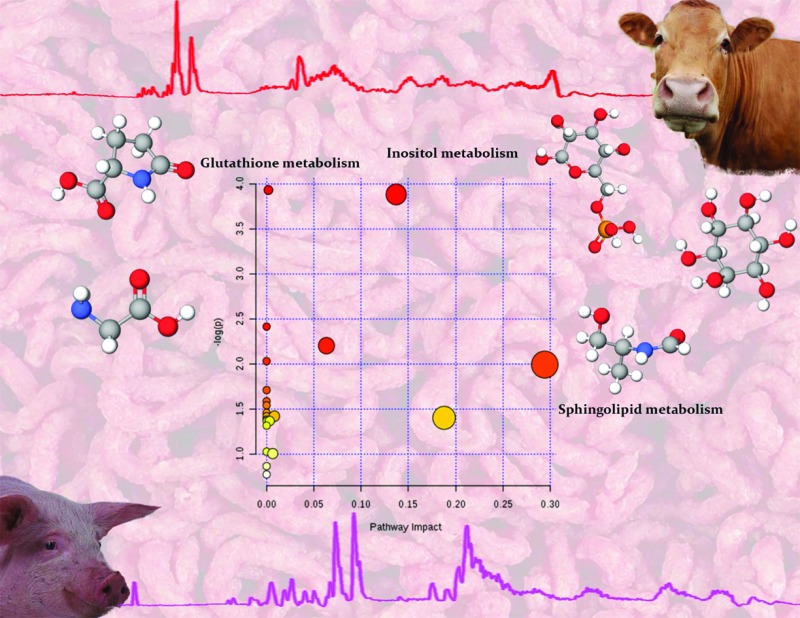
Adulteration of high quality food products with sub-standard and cheaper grades is a world-wide problem taxing the global economy.

## Introduction

Food fraud, also referred to as economically motivated adulteration (EMA),^1^ occurs when the intention is to deceive consumers deliberately by placing (usually) lower grade foodstuffs on the market for financial gain. This fraudulent activity can involve food products that are harmful, or unfit for human consumption, or deliberately mislabelled or misdescribed in some form.^2^ Whilst likely to be a problem as old as the food processing and production systems themselves,^3^ food fraud has very much become an emerging issue and one of great interest, especially so following large-scale events such as the melamine scandal in China,^4^ and the horsemeat crisis centred in the UK and Europe.^5^ These latter two events and many others over the last few decades^3^ have been said to be a result of globalisation and the ever increasing scale and complexity of food supply networks, which can lead to them becoming significantly more vulnerable to fraud and contamination, as well as being considered dysfunctional.^2^

In addition, meat has been said to be one of the most commonly consumed high value foods in the world,^6^ opening it up to fraudulent replacement/substitution of some, or all, of the premium meat content with lower grade cuts of meat or meat from other species, as was the case in the horsemeat crisis.^7^ The horsemeat crisis also demonstrated that minced meat is especially vulnerable to food fraud, since the original cut of meat is now visibly unrecognisable. In addition, the use of pork as an adulterant is of particular interest as it is a food eschewed by several groups internationally, for religious, moral, cultural, or dietary health considerations.^8^

Metabolomics is known as the “comprehensive analysis of the whole metabolome, which refers to the full complement of small molecule metabolites in a cell, tissue or organism, under a given set of conditions”.^9,10^ It is a relatively new area of science being used to gain a greater understanding of the chemical constituents and flux within biological systems. Metabolomics has predominantly been used in clinical^11^ and pharmaceutical research areas including work related to drug discovery^12^ and shows its versatility by being able to analyse a range of bio-fluids such as urine^13–15^ and serum/plasma^16^ as well as eukaryotic cells and microbes.^17,18^ However, the utility of this field has of course spurred interest in many areas of research, including food analysis, and indeed, some of the earliest publications within this field included the analysis of meat products, both for detection of microbial spoilage and shelf-life estimation^19,20^ and meat speciation as a first step toward detection of adulteration.^8,21^ With more recent reports of metabolomics studies of feed fraud^22^ and metabolomics for the detection of mechanically recovered meat.^23^ Perhaps not surprisingly, other omics approaches have also been used within the area of food analysis, such as proteomics in meat science,^24^ analysis of meat quality attributes such as tenderness,^25,26^ and multiple omics approaches for the elucidation of meat quality.^27^

It could be said that metabolomics allows for a far more complete and in-depth analysis into food composition and therefore can be utilised in the investigation into meat adulteration.^28^ Like human muscle tissue, a wide range of metabolites can be potentially found in animal tissue consumed as meat, therefore using metabolomics for the detection and discrimination of foreign meat species or indeed other contaminants^29–31^ in meat products is an area rich in potential. One of the most distinctive, and interesting, compositional element between two meat species is the content and type of fat, which is also linked to dietary health concerns, as well as being an important flavour component. Fat deposition, as well as consumption, is highly regulated by metabolism of the species and could potentially provide an insight into the discovery of biomarkers for identification of adulterated or contaminated meat.

Lipidomics is “the emerging field of systems-level analysis of lipids and factors that interact with lipids”^32^ and could be said to be a relatively unexplored area^33^ within food analytics and more specifically meat adulteration. Lipid profiling has been used previously for the chemical identification and differentiation of pathogenic bacteria, demonstrated in a large number of publications (*e.g.* ref. 34). Many lipids, such as fatty acids and triglycerides, have a high abundance in the metabolome^35^ and are the most distinctive of the biomarkers where each tissue type has a different lipid profile^34^ allowing them to be very useful in the identification of unwanted species present in food products. For each species of animal, there is a set amount of fatty acids located in the tissue specific to that species which can be used to distinguish between varying ‘foreign’ animal species found in a meat product and possibly used to quantify the amount of foreign meat species.^36^ However, another key advantage in lipidomics allows for the detection of the place of origin of the animal from which the sample was obtained.^37^ In this study, RPLC-MS was used for profiling differential lipids and GC-MS was used to investigate differential metabolites of primary metabolism, in order to identify and quantify the contamination of pork, in beef with different fat content.

## Materials

Fresh minced meat products were purchased from a national retail outlet. Pyridine (extra dry), hexane, methoxylamine hydrochloride, and *N*-methyl-*N*-trimethylsilyl-trifluoroacetamide (MSTFA) were obtained from Acros Organics (Loughborough, UK). The internal standards benzoic acid-*d*_5_, succinic acid-*d*_4_, and glycine-*d*_5_ were purchased from Sigma-Aldrich (Gillingham, UK). The solvents were all Optima LC-MS grade unless otherwise stated and purchased from Fisher Scientific (Gillingham, UK).

## Methods

### Sample collection

Minced beef and pork were purchased from a national retail outlet (labelling information listed in Table 1) and immediately stored at 4 °C until processed the following day. Four types of beef mince were purchased based on their fat content, which were: 5%, 15%, 20% and 23% fat respectively. Only one type of pork mince containing 5% fat was used in this study to keep variability to a minimum.

**Table 1:** Labelling details found on retail packaging of minced meats

Type of meat	Fat content (%)	Collagen/meat protein ratio (%)	Healthmark	Use by date	Date of purchase
Irish beef lean steak	5%	Less than 12%	UK 5416 EC	10 SEP 2015	9 SEP 2015
Irish beef steak mince	15%	Less than 15%	UK 5416 EC	12 SEP 2015	9 SEP 2015
Irish beef mince	20%	Less than 17%	UK 5416 EC	13 SEP 2015	9 SEP 2015
British beef mince	23%	Less than 18%	UK 8299 EC	13 SEP 2015	9 SEP 2015
British pork lean mince	5%	Less than 3%	UK 4175 EC	14 SEP 2015	9 SEP 2015

### Sample preparation

Aliquots (100 mg) of each mince were accurately weighed (±5 mg) into 2 mL Eppendorf tubes and transferred to –80 °C for storage. Tissue homogenisation and subsequent metabolite extraction was undertaken using a Tissuelyser II (Qiagen). The homogenisation solvent (1 : 1 chloroform : methanol, 800 μL pre-chilled to –20 °C) was added to each sample, a steel bead was then added and subsequently homogenised for 20 min at 25 Hz. Once homogenised, 400 μL of HPLC grade water was added and the sample vortex mixed for 15 s. To initiate phase separation the samples were then centrifuged (8000*g* for 10 min) before the aqueous and organic fractions were collected for GC-MS and RPLC-MS analysis respectively.

The ‘adulterated’ samples were then manually prepared by spiking the beef fractions with an appropriate volume of pork fraction, to levels of 0%, 10%, 25% and 50% adulteration with five biological replicates. Five minced “pork only” replicates were also prepared and all sample information is summarised in Table 2. The subsequent aqueous and organic fractions were lyophilised overnight (12 h), thus providing sample sets for both GC-MS and RPLC-MS analyses, respectively. These sample pellets were stored at –80 °C until required for analysis.

**Table 2:** Pork mince adulteration quantities

Meat	Adulterations (% of pork)	Number of samples (total 85)
Beef (5% fat)	0, 10, 25, 50	20 (4 samples × 5 replicates)
Beef (15% fat)	0, 10, 25, 50	20 (4 samples × 5 replicates)
Beef (20% fat)	0, 10, 25, 50	20 (4 samples × 5 replicates)
Beef (23% fat)	0, 10, 25, 50	20 (4 samples × 5 replicates)
Pork (5% fat)	—	5 (replicates)

### GC derivatization

Prior to GC-MS analysis, a two-stage chemical derivatization process was carried out to impart volatility to non-volatile metabolites, while also enabling thermal stability. These procedures are extensively described in ref. 38–40 and subsequent metabolite identification strictly followed the Metabolomics Standards Initiative guidelines as explained in ref. 41.

### GC-MS analysis and deconvolution

GC-MS analysis was carried out using an Agilent 6890N GC oven (Wokingham, UK) coupled to a Leco Pegasus III/IV mass spectrometer (St Joseph, USA) operated using ChromaTOF software v2.15. The GC oven used 6 N helium as the carrier gas, in a 3 : 1 split mode with a start temperature of 70 °C. A VF5-MS column (Supelco, Gillingham, UK, 30 m × 0.25 mm × 0.25 μm film thickness) was used with the transfer line and source temperatures held at 230 °C and 200 °C respectively. Samples were analysed within a mass range of 30–600 Da at a detector voltage of 1550 V. GC ramping and MS scan acquisition settings were identical to those used in ref. 42 and raw data were analysed, deconvolved with metabolites identified using the proprietary ChromaTOF software package as set out in ref. 43. All metabolite identifications were either MSI level 1 coming from our own in-house metabolite database, or MSI level 2 coming from the NIST version 8.0 database. The final output from this procedure was a retention time *vs.* mass data matrix with related metabolite IDs and peak areas linked to each sample injection. Peaks that had more than 50% missing values were removed and those that had more than 20% RSD within pooled QCs were also removed. Any further missing values were replaced by *k*-nearest neighbour algorithm. Data were normalised to total ion count, then log_10_ transformed and subsequently auto-scaled. This robust data set was then used for further statistical analysis.

### RPLC-MS analysis

Lyophilised sample pellets were reconstituted in LC-MS grade chloroform : methanol : water (1 : 4 : 4, v/v, 225 μL) and added to a LC-MS clear vial with a fixed insert. Analysis was carried out on an Accela UHPLC auto sampler system using a Hypersil Gold C_18_ reversed phase column (100 mm × 2.1 mm × 1.9 μm) coupled to an electrospray LTQ-Orbitrap XL hybrid mass spectrometry system (Thermo Fisher, Bremen, Germany) as previously described in ref. 39. Xcalibur and TunePlus software were used for instrument operation. Tuning and calibration was carried out as per the manufacturer's instruction. Five μL of each sample was injected on to the column and a methanol/water solvent gradient was used for metabolite separation on the stationary phase (Table 3). Note that both carrier solvents contained 0.1% formic acid to aid the ionization within the ESI source of the mass spectrometer. Samples were analysed in positive ESI modes using the following settings: 1 micro scan per 400 ms, 50–2000 *m*/*z* range, ESI ion source transfer tube set at 275 °C, tube lens voltage set at 100 V, capillary voltage set at 30 V, sheath gas flow rate set at 40 arbitrary units, auxillary gas flow set at 5 arbitrary units and sweep gas at 1 arbitrary units. Data were collected in profile mode at a mass resolution of 30 000.

**Table 3:** UHPLC-MS solvent gradient for reverse phase analysis

Time (min)	Flow rate (μL min^–1^)	Mobile phase A (H_2_O%)	Mobile phase B (MeOH%)
0	400	90	10
5	400	90	10
15	400	5	95
25	400	5	95
30	400	90	10

### RPLC-MS deconvolution and data processing pipeline

Initially, RAW data files were converted in to netCDF format (common data format – a generic code used for cross system comparisons *via* statistical platforms such as Matlab and R) within the software conversion option of Xcalibur and moved forward to deconvolution *via* the XCMS algorithm. Subsequently, our own in-house peak picking and deconvolution software written in R-code and utilizing the XCMS algorithm (; http://masspec.scripps.edu/xcms/xcms.php) was used. The resultant data matrix was retention time *vs*. mass and peak areas linked to each sample injection. Peaks that had more than 50% missing values were removed and those that had more than 20% RSD within pooled QCs were also removed. Any further missing values were replaced by *k*-nearest neighbour algorithm. Data were normalised to total ion count, log_10_ transformed and then auto scaled. This robust data set was then used for further statistical analysis. This included multivariate and univariate testing as detailed below. Data from this study are made available to download from Metabolights (; http://www.ebi.ac.uk/metabolights/studies).

## Results and discussion

**Fig. 1:**
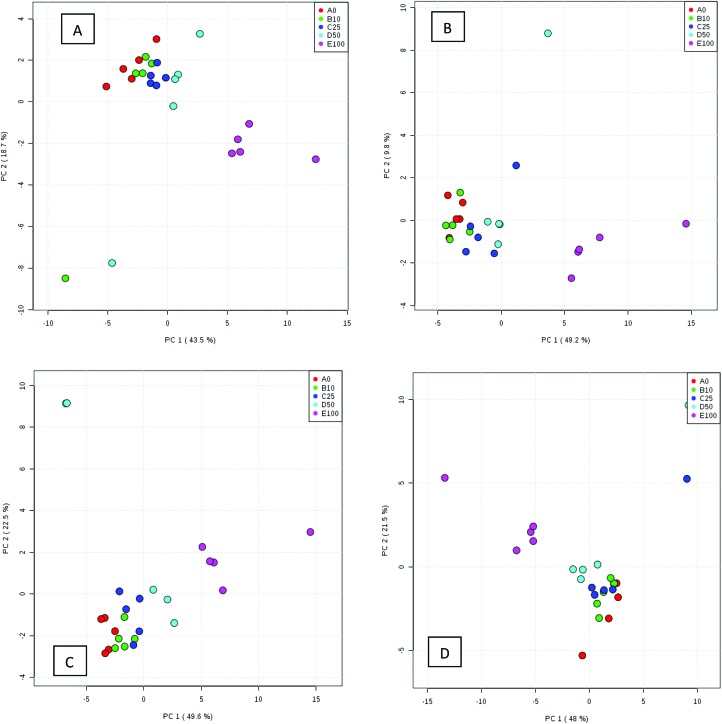
PCA scores plots for four beef types adulterated with different levels of pork: (A) beef containing 5% fat, (B) beef with 15% fat, (C) beef with 20% fat and (D) beef with 23% fat. Samples were analysed using GC-MS. The legends indicate the percentage of pork added to each beef type. Axes labels in parenthesis refer to the total explained variance.

**Fig. 2:**
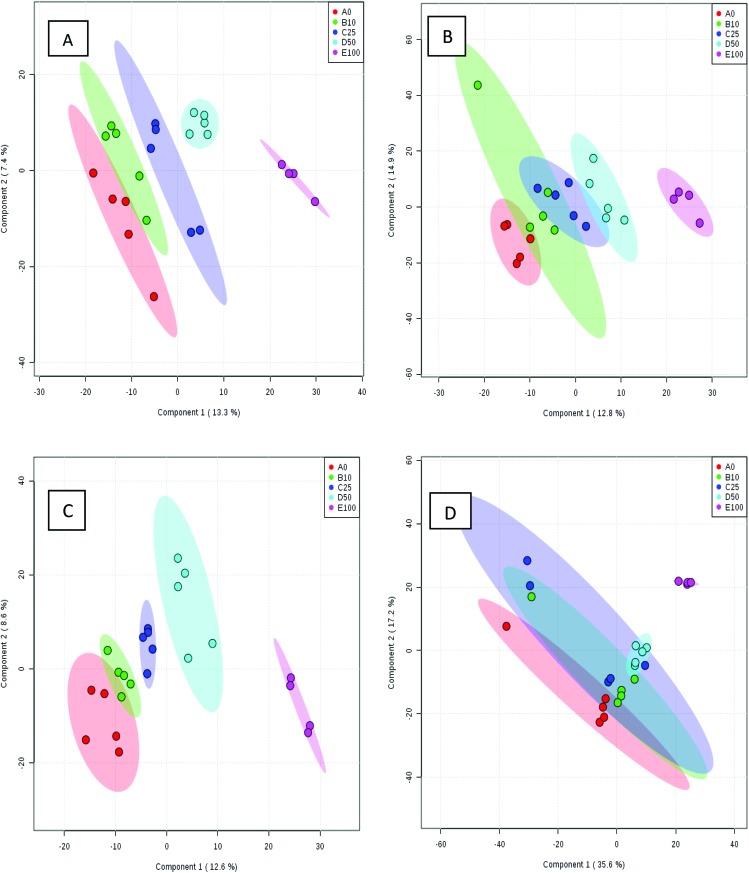
PLS-DA scores plots for four beef types adulterated with different amounts of pork: (A) beef containing 5% fat, (B) beef with 15% fat, (C) beef with 20% fat and (D) beef with 23% fat. Samples were analysed using LC-MS. The legends indicate the percentage of pork added to each beef type. Axes labels in parenthesis refer to the total explained variance.

Processed data were used for statistical analysis employing MetaboAnalyst version 3.0.^44^ Both GC-MS and LC-MS data were each divided into four constituent analysis groups based on their fat content in beef, in order to avoid the introduction of any variability of differential fat contents. Principal component analysis (PCA) and partial least squares discriminant analysis (PLS-DA) were performed and significant loadings were cross-confirmed by Kruskal–Wallis ANOVA (*p* < 0.05). Further variables were selected using Spearman's correlation analysis. PCA of GC-MS data showed a distinct gradient profile for increasing amount of pork adulteration in beef in the first principal component (PC1). This shows that there is quantitative information in these data as PC1 is extracted to explain the most natural variance and accounted for typically 50% in the four PCA scores plots. Pure beef and pure pork were clustered on the two extremes of a linear scattering with 10%, 25% and 50% pork contaminated beef located between these two clusters. This trend was observed for all beef types, irrespective of initial fat content of the beef (Fig. 1); note that the direction of the clustering (left to right or right to left) does not reflect anything statistically relevant as it is the trend not the direction that is important. These encouraging results were corroborated with PLS-DA result of LC-MS data (Fig. 2). Supervised multivariate analysis was required due to the higher complexity and feature-richness of LC-MS data compared to GC-MS data. It should be noted that, PCA for GC-MS explained more variance between adulteration content compared to PLS-DA for LC-MS data, despite both showing more within group variation.

**Fig. 3:**
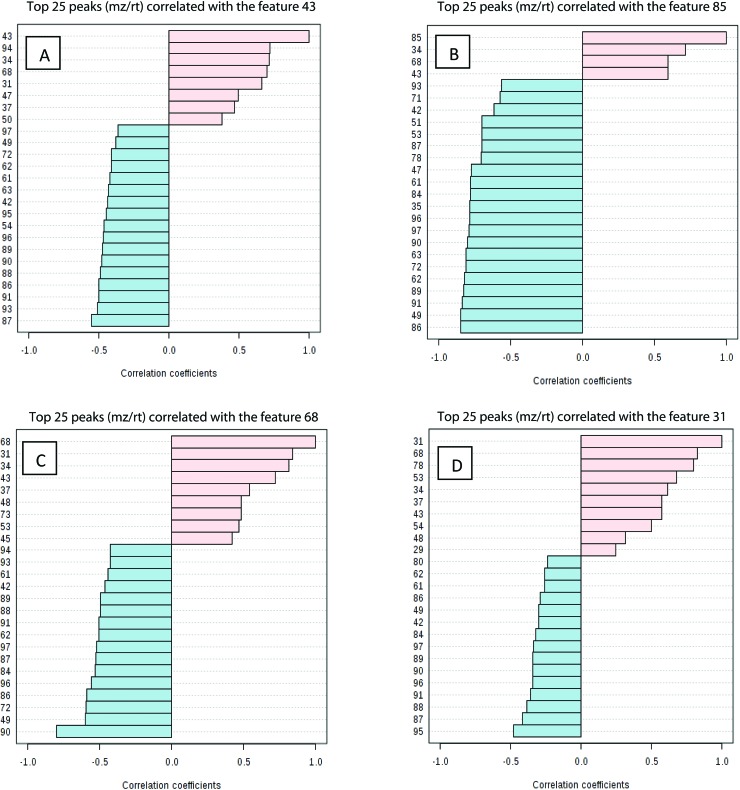
Pattern recognition – Spearman's correlation analysis showing the top 25 most correlated (positively and negatively) variables (metabolite features) with key loadings in type of beef. Each row represents the most significant variable identified from the test (*p* < 0.05). (A) beef with 5% fat, (B) beef with 15% fat, (C) beef with 20% fat and (D) beef with 23% fat. The *x*-axis shows correlation score whereas the *y*-axis corresponds to GC-MS peak number from peak index.

**Fig. 4:**
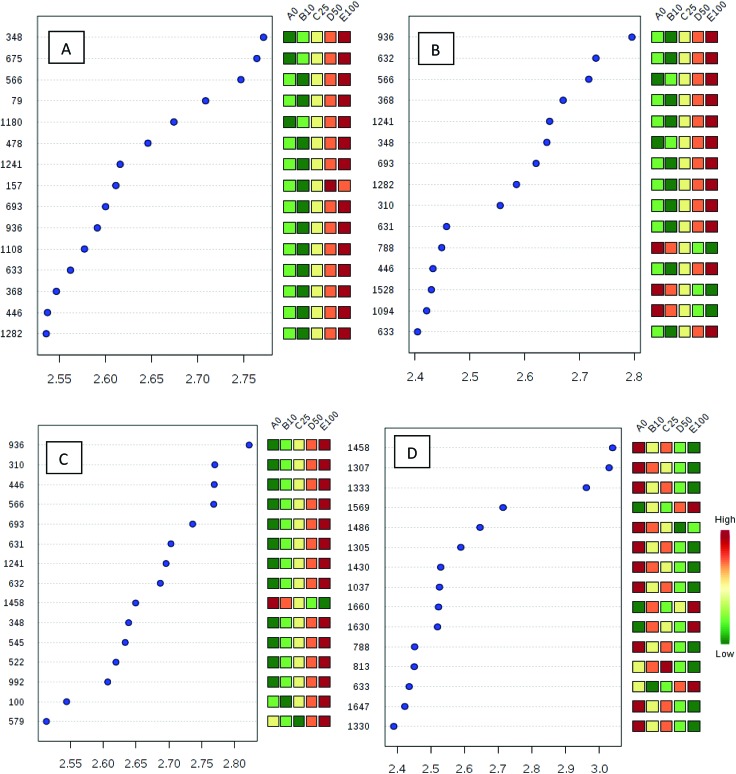
Top 15 metabolite features (variables) based on VIP scores from PLS-DA for each type of beef. (A) beef with 5% fat, (B) beef with 15% fat, (C) beef with 20% fat and (D) beef with 23% fat. The *x*-axis shows the correlation scores whereas the *y*-axis corresponds to the LC-MS peak number from peak index. Colour bars show median intensity of variable in the respective group.

**Fig. 5:**
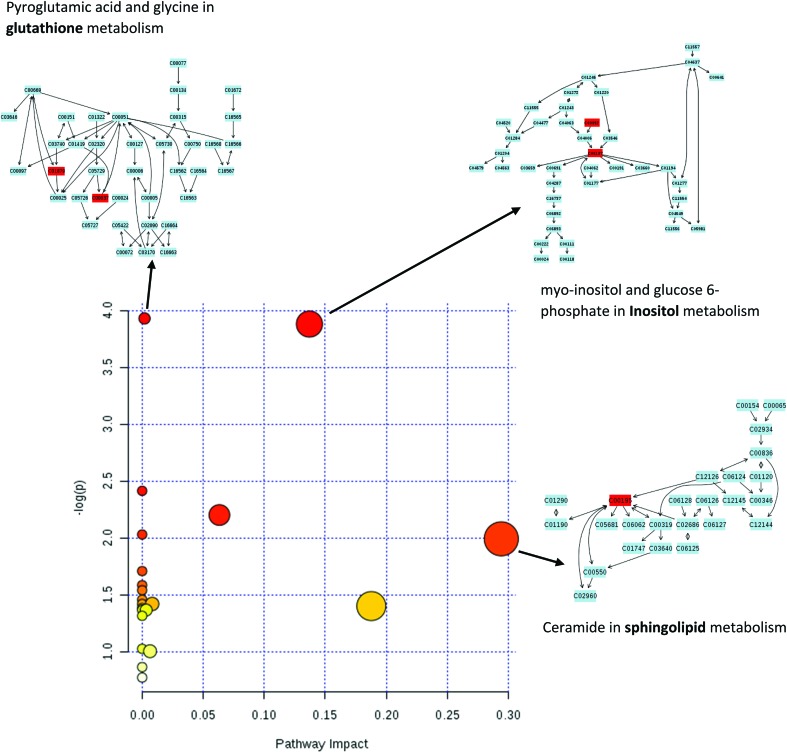
Pathway analysis using all the significant metabolites revealed significant differences in glutathione, inositol and sphingolipid metabolism between beef and pork mince. In the scatter plot the *x*-axis indicates impact on pathway whereas the *y*-axis indicates significant changes in a pathway, by detected metabolites (in red). Cxxxxx numbers in above pathways are identifiers for metabolites mapped in a KEGG pathway (accessible at ; http://www.genome.jp/kegg/pathway.html). Blocks in red indicate detected metabolites and blocks in blue are other metabolites present in a given pathway.

Studying the multivariate loadings vectors in each case (beef-pork at different fat contents, as well as GC-MS and UHPLC-MS) revealed various metabolites that increased in relative concentration with pork adulteration of beef mince. Kruskal–Wallis ANOVA was used to establish and confirm the significance of important loadings. Metabolites with patterns correlating to those of the most significant loadings were identified by performing Spearman's correlation analysis. Fig. 3 shows the top 25 highly correlating and significant metabolites (*p* < 0.05), that increase with increasing pork contamination of beef mince; these include both metabolites that increased when pork was added (shown in red; *i.e.* positive R) or decreased (highlighted in blue; negative R). Variable importance for prediction (VIP) scores were also calculated from the PLS-DA and Fig. 4 highlights the top 15 highly significant metabolites that were identified for each type of adulterated beef. Table 4, shows a list of all metabolites that were significantly correlated with pork adulteration of beef, irrespective of fat content and is the result of this combined PCA, PLS-DA and Kruskal–Wallis ANOVA. These analytes were used further to perform pathway analysis using MetaboAnalyst (Fig. 5). It was noted that glutathione and inositol pathways were probably the most differential pathways due to the amounts of pyroglutamic acid, *myo*-inositol, glucose 6-phosphate and glycine that allowed clear differentiation; between the two meat species, pork and beef. Increased glycine in pork could be indicative of slower glycine clearance in pigs than cattle. Glycine degradation occurs through three pathways: the glycine cleavage system, serine hydroxymethyltransferase, and conversion to glyoxylate by peroxisomal d-amino acid oxidase. The glycine cleavage system is the major enzyme system to initiate glycine degradation to form ammonia and CO_2_ in animals. Glycine is also utilized for the biosynthesis of glutathione, heme, creatine, nucleic acids, and uric acid by mammals.^45^ Increased glutathione metabolism in turn could lead to increased pyroglutamic acid, an intermediate metabolite of glutathione degradation.^46^ There is no evidence in the literature to our knowledge that indicates vast differences in glycine degradation between cattle and pigs, thus, lower glycine content in beef may also be suggestive of higher utilization in biosynthesis of energy metabolism regulating analytes in cattle due to higher demand of energy than pigs.^47^ Increased presence of glucose 6-phosphate in pork could be due to external intervention during meat preparation rather than animal metabolism. Glucose 6-phosphate, glucose, ribose and other monosaccharides are known to be added to pork to enhance its aroma and flavour in commercially available meat.^48^

**Table 4:** Unique significant metabolites detected by GC-MS and LC-MS. Only those that increased with percentage of pork contamination in beef mince are shown

Significant unique metabolites	Technique	MSI level	Identifier from relevant database
3-Oxohexadecanoic acid glycerides	LC-MS	2	N/A
Arabitol	GC-MS	1	CHEBI:22605
CE(22 : 5)	LC-MS	2	LMST01020031
Cer(d18 : 1/24 : 1)	LC-MS	2	LMSP02010009
Citric acid	GC-MS and LC-MS	1	CHEBI:30769
Creatinine	GC-MS	1	CHEBI:16737
Decanoylcholine	LC-MS	2	CHEBI:74107
Glucose 6-phosphate	GC-MS	2	CHEBI:17665
Glycine	GC-MS	1	CHEBI:15428
Glycyl-Lysine	LC-MS	2	CHEBI:73909
Heptadecane	GC-MS	1	CHEBI:16148
Hexano-dibutyrin	GC-MS	2	PUBCHEM:551329
Malic acid	GC-MS	1	CHEBI:6650
myo-inositol	GC-MS	1	CHEBI:17268
*N*-Carboxyethyl-*g*-aminobutyric acid	LC-MS	2	HMDB02201
Oleic acid	LC-MS	2	CHEBI:16196
Pentadecane	GC-MS	2	CHEBI:28897
PG(36 : 4)	LC-MS	2	N/A
Phosphate	GC-MS	1	HMDB01429
Prostaglandin D2 ethanolamide	LC-MS	2	CHEBI:85174
Pyroglutamic acid	GC-MS	1	HMDB00267
TG(16 : 0/15 : 0/18 : 4)	LC-MS	2	N/A
xi-2-Ethyl-1-hexanol	LC-MS	2	HMDB31231

Significant correlation between these set of analytes with pork mince could be key to monitor and detect levels greater than 10% adulteration of pork in beef mince. Furthermore, ceramide content between the two species of meat differed, indicating a strong impact on sphingolipid metabolism. Most foods of mammalian origin – for example beef, pork, dairy produce – have a variety of complex sphingolipids *e.g.* sphingomyelins, cerebrosides, globosides, gangliosides or sulfatides. These sphingolipids have many different head group components and ceramide backbones, hydrolysed during digestion in the lower colon.^49^

It should be noted here that the inherent complexity of metabolomics data in such research and its interpretation is not always straightforward. Animals (like humans) are fed on different diets in different countries. An obvious difference between pigs and cattle is that pigs are omnivores and cattle are herbivores having a more complex gut anatomy which may be reflected in the absorbance of carbon and nitrogen containing metabolites, many of a microbial origin. In addition to environmental factors *e.g.* climate, diet, source of diet, the exact method of meat preparation as well as storage, could greatly affect the measured metabolites for meat contamination or adulteration, and hence be reflected in the meat metabolome. As of course can any small molecules produced by bacteria, yeast or fungi that may be growing on the meat surface.^20,50,51^ Here, we forward a panel of metabolites, which we consider to have been successfully demonstrated as having a strong association with pork, and which increase conjointly with the increasing levels of pork added into beef mince. However, further investigation in variables that influence these signature metabolites is needed, to establish if their source is animal metabolism and not as a result of processes related to meat preparation/production. One example of this and a known key external factor in altered chemical contents of meat is irradiation.^52^ A process which is carried out in many large-scale meat production plants in order to alleviate the risk of bacterial infections and contamination. However, if not regulated, higher amount of irradiation could cause accelerated lipid oxidation, which in turn increases free fatty acid content.^53,54^

## Conclusion

Pork is of course inherently different to beef, in biochemical terms and also economically, with the partial substitution of the more expensive beef with cheaper pork an attractive proposition for those inclined to adulterate the food supply for economic gain. This practice is not generally life-threatening but can have profound religious, moral, cultural, or dietary health considerations.^20^ Here we have clearly demonstrated a panel of metabolites linked directly to pork and shown that these increase in line with the levels of adulteration of beef mince with pork. It would take further investigation to establish whether all of these metabolites are linked to animal metabolism or are a consequence of meat production processes of these two species. In addition, sphingolipid metabolism whilst higher in pork, could also suggest excessive irradiation that increases free fatty acids. A further targeted fatty acid assay could not only confirm this but also provide a measure of excessive irradiation of meat, for regulation purposes. There was also a subset of metabolites which appeared to be directly correlated to the level of fat content.

We believe that the application of omics technologies (such as illustrated here by the range of advanced hyphenated mass spectrometry used in this study), to the challenges of food adulteration and labelling, would aid in the identification and quantification of small molecule/metabolite targets. These molecules could, after further validation, then act as food resilience biomarkers, the detection of which does not necessarily need complex mass spectrometry but could be readily achieved by targeted chromatography or specific chemical sensing approaches. These could have the potential to serve as early diagnostic warning systems, with one, or combination of these markers related to specific forms of problems or behaviours/activities within food processing, production or supply systems. This could allow for early intervention and corrective, preventative, or regulatory action within food supply chains, as well as the ability to learn from any information that these markers may provide, in order to recognise and deter/disrupt future negative impacts (such as food fraud) from a variety of potential sources.
